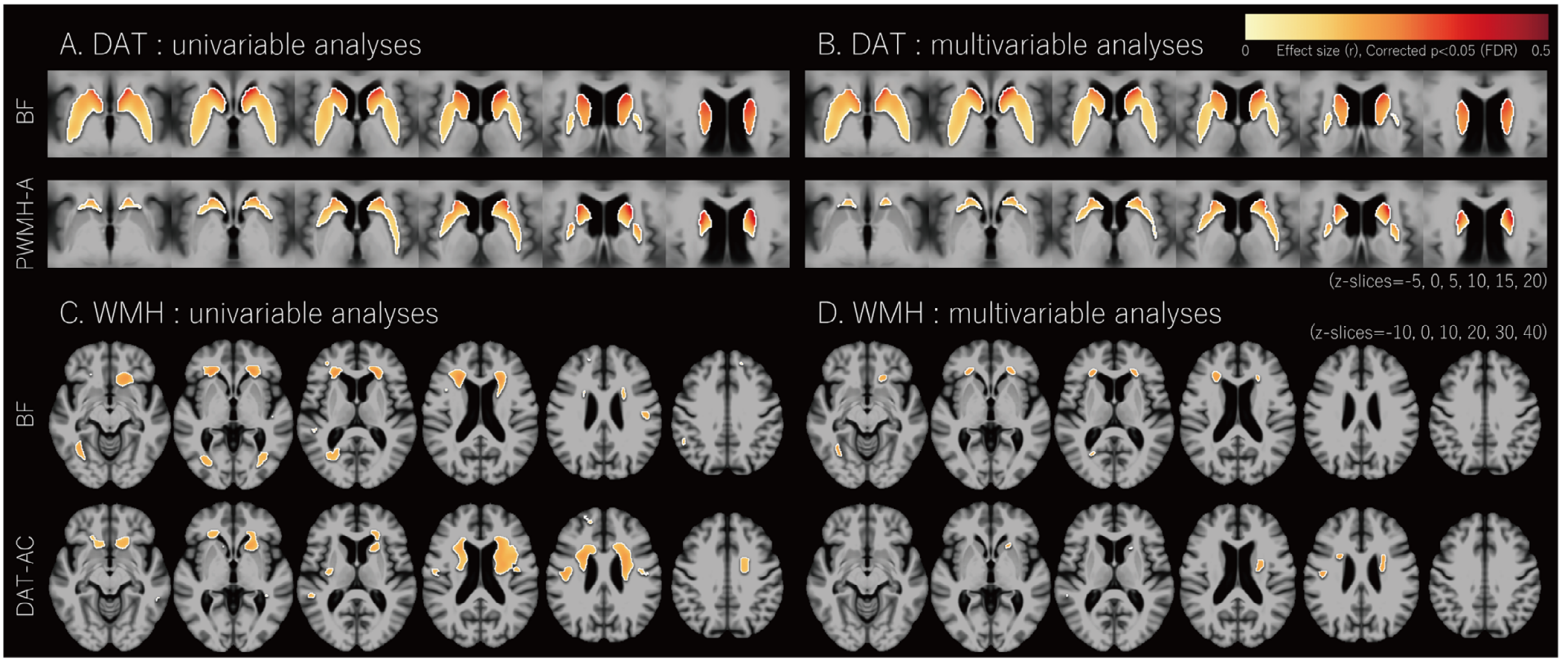# White Matter Hyperintensities and Cholinergic Degeneration as Lewy Body Disease

**DOI:** 10.1002/alz.088424

**Published:** 2025-01-09

**Authors:** Sungwoo Kang, Seun Jeon, Byoung Seok Ye

**Affiliations:** ^1^ Yonsei University College of Medicine, Seoul Korea, Republic of (South); ^2^ Brain Research Institute, Yonsei University College of Medicine, Seoul Korea, Republic of (South)

## Abstract

**Background:**

To investigate the relationship between basal forebrain (BF) cholinergic activity, dopaminergic degeneration, white matter hyperintensities (WMHs), and their effects on clinical manifestations of Alzheimer’s disease (AD) and Lewy body disease (LBD).

**Method:**

A total of 407 subjects who underwent 3‐T MRI, dopamine transporter (DAT) positron emission tomography, neuropsychological tests, and assessments for parkinsonism, cognitive fluctuation (CF), visual hallucination (VH), and rapid eye movement sleep behavior disorder (RBD) were evaluated for probable AD, LBD, or both (AD+LBD). General linear models were used to investigate the relationships between BF volume (BFV), striatal DAT uptake, WMHs, and clinical manifestations after controlling for age, sex, education, vascular factors, and intracranial volume. Quantitative analyses of DAT uptake in the anterior caudate (AC), posterior caudate (PC), anterior putamen (AP), and posterior putamen (PP) and assessment of WHMs in the anterior/posterior periventricular (PWMH‐A/PWMH‐P) and deep regions (DWMH‐A/DWMH‐P) were performed.

**Result:**

DAT‐AC was positively associated with BFV. DAT‐AC was negatively associated with PWMH‐A, but not with DWMHs, independent of BFV. Both DWMHs and PWMHs were associated with hypertension and the number of microbleeds and lacunae. Lower BFV and DAT‐AC were independently associated with increased risk of CF and VH, whereas lower DAT‐AC was additionally associated with an increased risk of RBD and higher parkinsonian severity. Lower BFV and DAT‐AC were independently associated with widespread cognitive impairment, whereas higher PWMH‐A was associated with executive dysfunction.

**Conclusion:**

Cholinergic degeneration in the BF and PWMH‐A levels are closely associated with dopaminergic degeneration. PWMH‐A may be a manifestation of axonal alterations caused by the interplay between LB‐related degeneration and vascular pathologies.